# Synergic Effect of Early Administration of Probiotics and Adipose-Derived Mesenchymal Stem Cells on Alleviating Inflammation-Induced Chronic Neuropathic Pain in Rodents

**DOI:** 10.3390/ijms231911974

**Published:** 2022-10-09

**Authors:** Kuan-Hung Chen, Hung-Sheng Lin, Yi-Chen Li, Pei-Hsun Sung, Yi-Ling Chen, Tsung-Cheng Yin, Hon-Kan Yip

**Affiliations:** 1 Department of Anesthesiology, Kaohsiung Chang Gung Memorial Hospital and Chang Gung University College of Medicine, Kaohsiung 83301, Taiwan; 2 Division of Neurology, Department of Internal Medicine, Kaohsiung Chang Gung Memorial Hospital and Chang Gung University College of Medicine, Kaohsiung 83301, Taiwan; 3 Center of Cell Therapy, National Cheng Kung University Hospital, College of Medicine, National Cheng Kung University, Tainan 70403, Taiwan; 4 Division of Cardiology, Department of Internal Medicine, Kaohsiung Chang Gung Memorial Hospital and Chang Gung University College of Medicine, Kaohsiung 83301, Taiwan; 5 Department of Orthopaedic Surgery, Kaohsiung Chang Gung Memorial Hospital and Chang Gung University College of Medicine, Kaohsiung 83301, Taiwan; 6 Institute for Translational Research in Biomedicine, Kaohsiung Chang Gung Memorial Hospital, Kaohsiung 83301, Taiwan; 7 Center for Shockwave Medicine and Tissue Engineering, Kaohsiung Chang Gung Memorial Hospital, Kaohsiung 83301, Taiwan; 8 School of Medicine, College of Medicine, Chang Gung University, Taoyuan 33302, Taiwan; 9 Department of Medical Research, China Medical University Hospital, China Medical University, Taichung 40402, Taiwan; 10 Department of Nursing, Asia University, Taichung 41354, Taiwan; 11 Division of Cardiology, Department of Internal Medicine, Xiamen Chang Gung Hospital, Xiamen 361028, China

**Keywords:** neuropathic pain, chronic constriction injury, probiotics, adipose-tissue-derived mesenchymal stem cells

## Abstract

This study investigated the hypothesis that probiotics enhanced the therapeutic effect of adipose-derived mesenchymal stem cells (ADMSCs) on alleviating neuropathic pain (NP) due to chronic constriction injury (CCI) mainly through regulating the microbiota in rats. SD rats (n = 50) were categorized into group 1 (sham-control), group 2 (NP), group 3 (NP + probiotics (i.e., 1.5 billion C.F.U./day/rat, orally 3 h after NP procedure, followed by QOD 30 times)), group 4 (NP + ADMSCs (3.0 × 10^5^ cells) 3 h after CCI procedure, followed by QOD six times (i.e., seven times in total, i.e., mimic a clinical setting of drug use) and group 5 (NP + probiotics + ADMSCs (3.0 × 10^5^ cells)) and euthanized by day 60 after NP induction. By day 28 after NP induction, flow-cytometric analysis showed circulating levels of early (AN-V^+^/PI^−^) and late (AN-V^+^/PI^+^) apoptotic, and three inflammatory (CD11b-c+, Ly6G+ and MPO+) cells were lowest in group 1 and significantly progressively reduced in groups 2 to 5 (all *p* < 0.0001). By days 7, 14, 21, 28, and 60 after CCI, the thresholds of thermal paw withdrawal latency (PWL) and mechanical paw withdrawal threshold (PWT) were highest in group 1 and significantly progressively increased in groups 2 to 5 (all *p* < 0.0001). Numbers of pain-connived cells (Nav1.8+/peripherin+, p-ERK+/peripherin+, p-p38+/peripherin+ and p-p38+/NF200+) and protein expressions of inflammatory (p-NF-κB, IL-1ß, TNF-α and MMP-9), apoptotic (cleaved-caspase-3, cleaved-PARP), oxidative-stress (NOX-1, NOX-2), DNA-damaged (γ-H2AX) and MAPK-family (p-P38, p-JNK, p-ERK1/2) biomarkers as well as the protein levels of Nav.1.3, Nav.1.8, and Nav.1.9 in L4-L5 in dorsal root ganglia displayed an opposite pattern of mechanical PWT among the groups (all *p* < 0.0001). In conclusion, combined probiotic and ADMSC therapy was superior to merely one for alleviating CCI-induced NP mainly through suppressing inflammation and oxidative stress.

## 1. Introduction

It is well recognized that neuropathic pain (NP) remains one of the most common chronic neurological disorders in patients who repeatedly seek medical help at hospitals, resulting in great economic burdens worldwide [1,2,3,4,5]. Numerous investigations have identified that NP resulting from traumatic accident and different disease entities influencing the peripheral nervous system, dorsal root ganglion/dorsal root, or central nerve system, constantly affects patients’ diathesis of life and functional condition or can even bring about psycho-social incapacitation [1,3,4,5]. Surely, an inappropriate sensitization of central and peripheral nervous systems is the leading cause of NP [1]. Additionally, emotional change will always exacerbate this sensation.

Despite advanced pharmaceutical and non-pharmaceutical efforts in improving NP, such as physical therapy, acupuncture therapy, anticonvulsant [6] and antidepressant agents [7], nerve blocks [8], and bioelectrical therapy [9,10], a large portion of patients are still unsatisfied with the treatment since the intractable and persistent pain is constantly recurrent, implying that current treatment of NP leaves much to be desired. Accordingly, to resolve this issue, the current mission is to develop an innovative therapeutic management with an innocuous and long-term interval of effectiveness.

The pathophysiologic mechanism participating in the NP is quite complicated, especially since it involves the malfunction of the central and peripheral nervous systems [11,12,13]. The processes of oxidative stress, neuro-inflammation, inflammatory cell and cytokine generation, and immune cell participation (i.e., innate immune system influences the adaptive immune responses) have been shown as the major molecular–cellular contributors of NP [13,14,15,16,17], which may account for why traditional therapy frequently failed to cure or control the symptoms/signs of NP.

An association between changing gut microbiota and chemotherapy-induced peripheral neuropathy has been thoroughly introduced [18,19]. Additionally, some studies have indicated that the gut microbiome is a key regulator of visceral pain and that the gut microbiota may also display a key role in a lot of other kinds of chronic pain, including inflammatory pain, headache, neuropathic pain, and opioid tolerance [20,21]. Interestingly, the crosstalk between alternation of gut microbiota and immune dysregulation has also been clearly identified by growing data [22,23].

Surprisingly, while the gut microbiota has been identified as playing a crucial role in the development of behavioral and cognitive dysfunctions, inflammatory reaction, immune dysregulation, metabolic disorder and neuropathic pain [18,19,20,21,22,23], effective strategic management has regrettably seldom been reported [24]. This pushes us to consider a new and safe therapeutic targeting the gut microbiota for the treatment of NP.

Mesenchymal stem cells (MSCs), particularly adipose-derived MSCs (i.e., ADMSCs), have been clearly identified to possess the intrinsic ability to ameliorate inflammatory reaction [25,26,27] and inhibit innate and adaptive immunity [25,26,27,28] through reducing immunogenicity [25,26,27]. Additionally, abundant evidence has clearly demonstrated that MSCs have the capacity to manage antioxidative stress, perform angiogenesis, and repair damaged tissue and organs, resulting in tissue/organ regeneration as well as improvement of mitochondrial dysfunction [25,26,27,29,30]. Additionally, even allogenic MSCs have been applied for patients with severe cardiovascular disease [31] or acute respiratory distress syndrome [32], and the results were reported to be safe and to have potentially improved the clinical outcomes. These findings [25,26,27,29,30,31,32] highlight that MSC therapy may possess therapeutic potential for NP patients. However, whether MSC therapy would provide benefits for NP is currently unclear. Probiotics conceal microorganisms, the majority of which are bacteria, such as the beneficial bacteria that develop naturally in the human gut. Probiotics have been largely investigated for various gastrointestinal diseases [33,34,35]. Most investigated species include *Lactobacillus*, *Bifidobacterium*, and *Saccharomyces* [33,34]. Probiotics have been identified as playing a principal role in maintaining immunologic equilibrium in the gastrointestinal tract via direct interplay with immune cells [33,34,35].

Growing investigations have demonstrated that probiotics act in a potentially therapeutic role in the treatment of breast cancer [36], inflammatory bowel diseases [37] and luminal gastrointestinal disorders [38], prevention of gestational diabetes mellitus (DM) [39], irritable bowel syndrome [40], allergic diseases [41], and even Parkinsonism [42]. However, whether probiotic therapy is also effective for NP is seldom reported [24]. Surprisingly, whether a combination of probiotic and ADMSC therapy would offer additional benefit on ameliorating the NP has not yet been investigated. In summary, the aforementioned issues [18,19,20,21,22,23,24,25,26,27,28,33,34,35,36,37,38,39,40,41,42] raise the hypothesis that probiotic therapy might enhance the effect of autologous ADMSCs on treatment of the chronic peripheral NP.

## 2. Results

### 2.1. The Rationale for Using Human Probiotics for Rodents in the Present Study

Recent reports indicated fecal microbial community of rats is more similar to human than mice [43,44]. Based on these aforementioned reports [43,44], we preliminarily evaluated the fecal microbiota of SD healthy rats (n = 2) and performed a similarity study with those of healthy human individuals (n = 5). From microbiome analysis, bacteroides accounted for 39% and dominated in the human microbiome; Prevotella accounted for 43% and dominated in the rat microbiome (Supplementary Table S1, Supplementary Figure S1). It is well recognized that *Lactobacillus* species are generally regarded as safe probiotics with a long history and a wide variety of applications. Although the preliminary data in Supplementary Table S1 and Supplementary Figure S1 showed that dominant microbiota were different between humans and rats, the percentages of *Lactobacillales* species were similar—3.1% in humans and 2.8% in rats. Accordingly, probiotics (PNT_BIO_-RAY^TM^ containing L.paracasei; Kao An Biomedical Co., Ltd., Kaohsiung, Taiwan) were used for the investigated rodents (Supplementary Table S1, Supplementary Figure S1).

### 2.2. Flow Cytometry Investigation for Circulatory Levels of Mononuclear Cell Apoptosis and Inflammatory Cells by Day 28 after NP Induction

**Figure 1:**
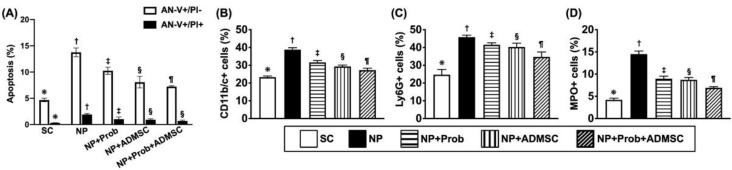
Flow cytometric analysis for circulatory levels of mononuclear cell apoptosis and inflammatory cells by day 28 after neuropathic pain (NP) induction. (**A**) Circulatory number of early apoptosis (AN-V^+^/PI^−^) of mononuclear cells, * vs. other groups with different symbols (†, ‡, §, ¶), *p* < 0.0001. Circulating level of late apoptosis (AN-V^+^/PI^+^) of mononuclear cells, * vs. other groups with different symbols (†, ‡, §), *p* < 0.0001. (B) Circulating number of CD11b/c+ cells, * vs. other groups with different symbols (†, ‡, §, ¶), *p* < 0.0001. (C) Circulating number of Ly6G+ cells, * vs. other groups with different symbols (†, ‡, §, ¶), *p* < 0.0001. (D) Circulating number of myeloperoxidase (MPO)+ cells, * vs. other groups with different symbols (†, ‡, §), *p* < 0.0001. Symbols (*, †, ‡, §, ¶) indicate significance (at 0.05 level). All statistical analyses were performed using one-way ANOVA followed by Bonferroni multiple comparison post hoc test (n = 4 for each group). SC = sham control; ADMSCs = adipose-tissue-derived mesenchymal stem cells.

The result demonstrated that the circulating levels of CD11b/c+ (Figure 1B), Ly6G+ (Figure 1C) and MPO+ (Figure 1D) cells, three indicators of inflammatory reaction [45] were lowest in group 1 (SC), highest in group 2 (NP only), and significantly lower in group 5 (NP + probiotics + ADMSCs) than in group 3 (NP + probiotics) and group 4 (NP + ADMSCs), but they showed similarly between groups 3 and 4, except for in CD11b/c+ cells, which were significantly lower in group 4 than in group 3.

Furthermore, the flow cytometric analysis demonstrated that the early apoptosis (i.e., annexin V+/propidium iodide solution (PI)−) (Figure 1A) of mononuclear cells was lowest in group 1, highest in group 2, and significantly progressively reduced in groups 3 to 5. On the other hand, the late apoptosis (i.e., annexin V+/propidium iodide solution (PI)+) (Figure 1A) of mononuclear cells was significantly lower in group 1 than in other groups and significantly lower in groups 4 and 5 than in groups 2 and 3, but it showed no significant difference between groups 4 and 5 or between groups 2 and 3. These parameters implied that early treatment with probiotics or ADMSCs, especially by combination of probiotics and ADMSCs treatment, remarkably suppressed NP-induced inflammatory reaction and cellular apoptosis.

### 2.3. Time Points of TPWL (Sec) and MPWT (g) in Rat

**Figure 2:**
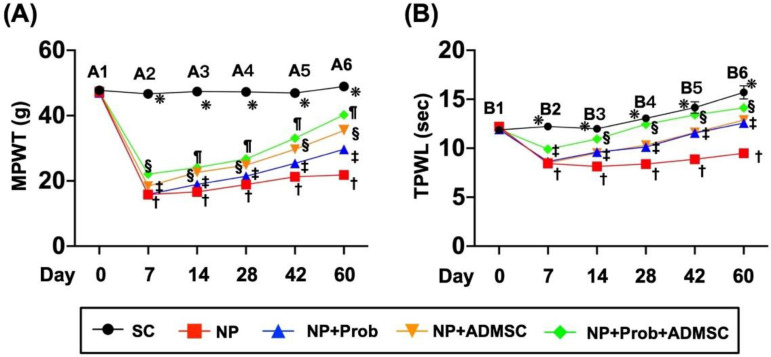
Time points of mechanical paw withdrawal threshold (MPWT) (g) and thermal paw withdrawal latency (TPWL) (Sec) in rats. (**A**) Left Panel: (A1) At day 0, the analytical results of MPWT were similar among the five groups. (A2) At day 7, the analytical results of MPWT, * vs. other groups with different symbols (†, ‡, §, ¶), *p* < 0.0001. (A3) At day 14, the analytical results of MPWT, * vs. other groups with different symbols (†, ‡, §, ¶), *p* < 0.0001. (A4) At day 28, the analytical results of MPWT, * vs. other groups with different symbols (†, ‡, §, ¶), *p* < 0.0001. (A5) At day 42, the analytical results of MPWT, * vs. other groups with different symbols (†, ‡, §, ¶), *p* < 0.0001. (A6) At day 60, the analytical results of MPWT, * vs. other groups with different symbols (†, ‡, §, ¶), *p* < 0.0001. (**B**) Right Panel: (B1) At day 0, the analytical results of TPWL were similar among the five groups. (B2) At day 7, the analytical results of TPWL, * vs. other groups with different symbols (†, ‡, §), *p* < 0.0001. (B3) At day 14, the analytical results of TPWL, * vs. other groups with different symbols (†, ‡, §), *p* < 0.0001. (B4) At day 28, the analytical results of TPWL, * vs. other groups with different symbols (†, ‡, §), *p* < 0.0001. (B5) At day 42, the analytical results of TPWL, * vs. other groups with different symbols (†, ‡, §), *p* < 0.0001. (B6) At day 60, the analytical results of TPWL, * vs. other groups with different symbols (†, ‡, §), *p* < 0.0001. Symbols (*, †, ‡, §, ¶) indicate significance for each other (at 0.05 level). All statistical analyses were performed using one-way ANOVA, followed by Bonferroni multiple comparison post hoc test (n = 10 for each group). SC = sham control; NP = neuropathic pain; Prob = probiotics; ADMSCs = adipose-tissue-derived mesenchymal stem cells; TPWL = thermal paw withdrawal latency; MPWT = mechanical paw withdrawal threshold.

By days 7 (Figure 2-A2), 14 (Figure 2-A3), 28 (Figure 2-A4), 42 (Figure 2-A5), and 60 (Figure 2-A6) after NP induction, the MPWT, the cardinal test for verifying the degree of NP, was highest in group 1, lowest in group 2, significantly higher in group 5 than in groups 3 and 4, and significantly higher in group 4 than in group 3, suggesting additional benefits of probiotics on ADMSC therapy in ameliorating NP in rodents. Additionally, the TPWL, another cardinal test for verifying the degree of NP, also exhibited a similar pattern to MPWT at the time points of days 7 (Figure 2-B2), 14 (Figure 2-B3), 28 (Figure 2-B4), 42 (Figure 2-B5), and 60 (Figure 2-B6), except for groups 3 and 4, which showed no difference between these time points.

### 2.4. Protein Levels of Inflammation and Oxidative Stress in Ipsilateral L4-L5 Dorsal Root Ganglia (DRGs) by Day 60 after NP Induction

**Figure 3:**
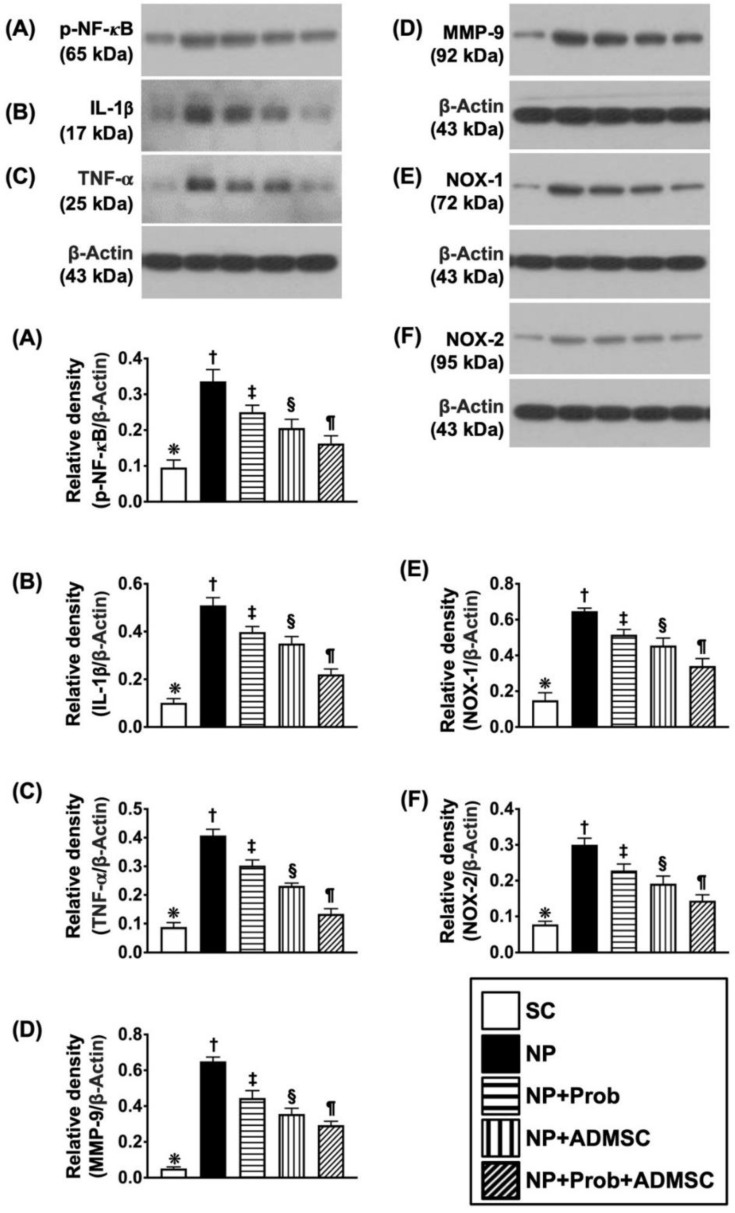
Protein expressions of inflammatory and oxidative stress biomarkers in ipsilateral L4-L5 dorsal root ganglia (DRGs) by day 60 after NP induction. (**A**) Protein expression of phosphorylated nuclear factor kapa B (p-NF-κB), * vs. other groups with different symbols (†, ‡, §, ¶), *p* < 0.0001. (**B**) Protein expression of interleukin (IL)-1β, * vs. other groups with different symbols (†, ‡, §, ¶), *p* < 0.0001. (**C**) Protein expression of tumor necrosis factor alpha (TNF-α), * vs. other groups with different symbols (†, ‡, §, ¶), *p* < 0.0001. (**D**) Protein expression of matrix metalloproteinase (MMP)-9, * vs. other groups with different symbols (†, ‡, §, ¶), *p* < 0.0001. (**E**) Protein expression of NOX-1, * vs. other groups with different symbols (†, ‡, §, ¶), *p* < 0.0001. (**F**) Protein expression of NOX-2, * vs. other groups with different symbols (†, ‡, §, ¶), *p* < 0.0001. Symbols (*, †, ‡, §, ¶) indicate significance for each other (at 0.05 level). All statistical analyses were performed using one-way ANOVA followed by Bonferroni multiple comparison post hoc test (n = 6 for each group). SC = sham control; NP = neuropathic pain; Prob = probiotics; ADMSCs = adipose-tissue-derived mesenchymal stem cells.

To elucidate the therapeutic effect of probiotics-ADMSCs on inhibiting the inflammatory reaction and oxidative stress in DRGs, the Western blot assessment was utilized in the current study. The result showed that p-NF-κB (Figure 3A), IL-1ß (Figure 3B), TNF-α (Figure 3C) and MMP-9 (Figure 3D), four indicators of inflammation [14,15,25,26,27], and NOX-1 (Figure 3E) and NOX-2 (Figure 3F), two indicators of oxidative stress [14,15,25,26,27], were lowest in group 1, highest in group 2, and significantly progressively reduced in groups 3 to 5.

### 2.5. Protein Levels of Apoptosis and DNA-Injured Markers in L4-L5 DRGs by Day 60 after NP Induction

**Figure 4:**
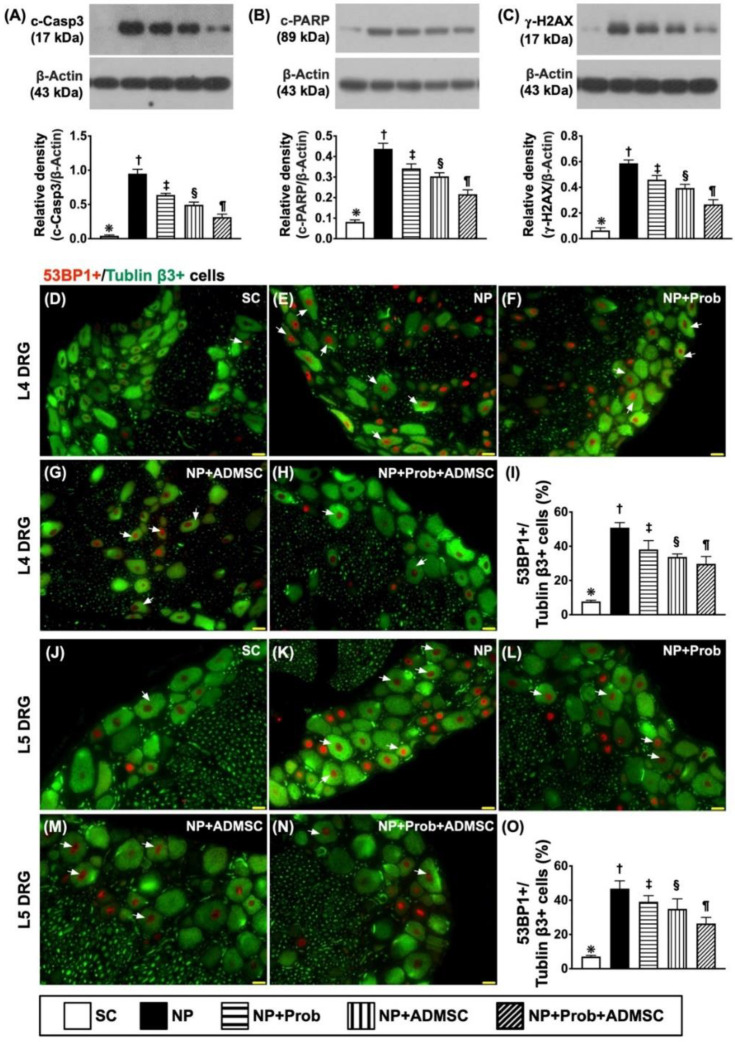
Protein expressions of apoptosis and DNA-damaged biomarkers and cellular expression of DNA damage in L4-L5 DRGs by day 60 after NP induction. (**A**) Protein expression of cleaved caspase 3 (c-Casp3), * vs. other groups with different symbols (†, ‡, §, ¶), *p* < 0.0001. (**B**) Protein expression of cleaved PARP1 poly(ADP-ribose) polymerase (c-PARP), * vs. other groups with different symbols (†, ‡, §, ¶), *p* < 0.0001. (**C**) Protein expression of γ-H2AX, * vs. other groups with different symbols (†, ‡, §, ¶), *p* < 0.0001. L4 DRG level: (**D**–**H**) Showing the immunofluorescent (IF) microscopic findings (400×) to identify merged (red–green co-localization), positively-stained 53PB1 (red color), and Tubulin β3 (green color). Note that the white arrows indicated the merged double stain of 53PB1+/Tubulin β3+. (**I**) Analytical results of number (%) of 53PB1+ cells compared to total Tubulin β3+ cells, * vs. other groups with different symbols (†, ‡, §, ¶), *p* < 0.0001. (n = 6 for each group). L5 DRG level: (**J**–**N**) Showing IF microscopic findings (400×) to identify merged (red-green co-localization), positively-stained 53PB1 (red color), and Tubulin β3 (green color). Note that the white arrows indicate the merged double stain of 53PB1+/Tubulin β3+. (**O**) Analytical results of number (%) of 53PB1+ cells compared to total Tubulin β3+ cells, * vs. other groups with different symbols (†, ‡, §, ¶), *p* < 0.0001. Scale bars in the lower-right corner represent 20 µm. Noted that positively stained 53PB1 indicates DNA-damaged markers, whereas the Tubulin β3 indicates the microtubule element of the tubulin in neurons. Symbols (*, †, ‡, §, ¶) indicate significance for each other (at 0.05 level). All statistical analyses were performed using one-way ANOVA followed by Bonferroni multiple comparison post hoc test (n = 8 for each group). SC = sham control; NP = neuropathic pain; Prob = probiotics; ADMSCs = adipose-tissue-derived mesenchymal stem cells.

The result of Western blot analysis showed that the protein expressions of cleaved caspase 3 (Figure 4A) and cleaved PARP (Figure 4B), two indicators of apoptosis [14,15,25,26,27], were lowest in group 1, highest in group 2, and significantly progressively reduced in groups 3 to 5. Additionally, the protein and cellular levels of γ-H2AX (Figure 4C), an indicator of DNA-damaged markers [14,15,25,26,27], displayed an identical pattern of apoptosis among the groups.

Furthermore, to elucidate whether the cellular level of DAN damage biomarker also consistently expressed the protein level in regions of L4-L5 DRGs, an IF microscope was utilized in the current study. As expected, the cellular expression of 53PB1/Tubulin β3 (i.e., indicated the double stain of 53PB1/Tubulin β3) (Figure 4D–O) exhibited an identical pattern to γ-H2AX.

### 2.6. The Protein Levels of MAPK Family in L4-L5 DRGs and Voltage-Gated Sodium Channels in Sciatic Nerve

**Figure 5:**
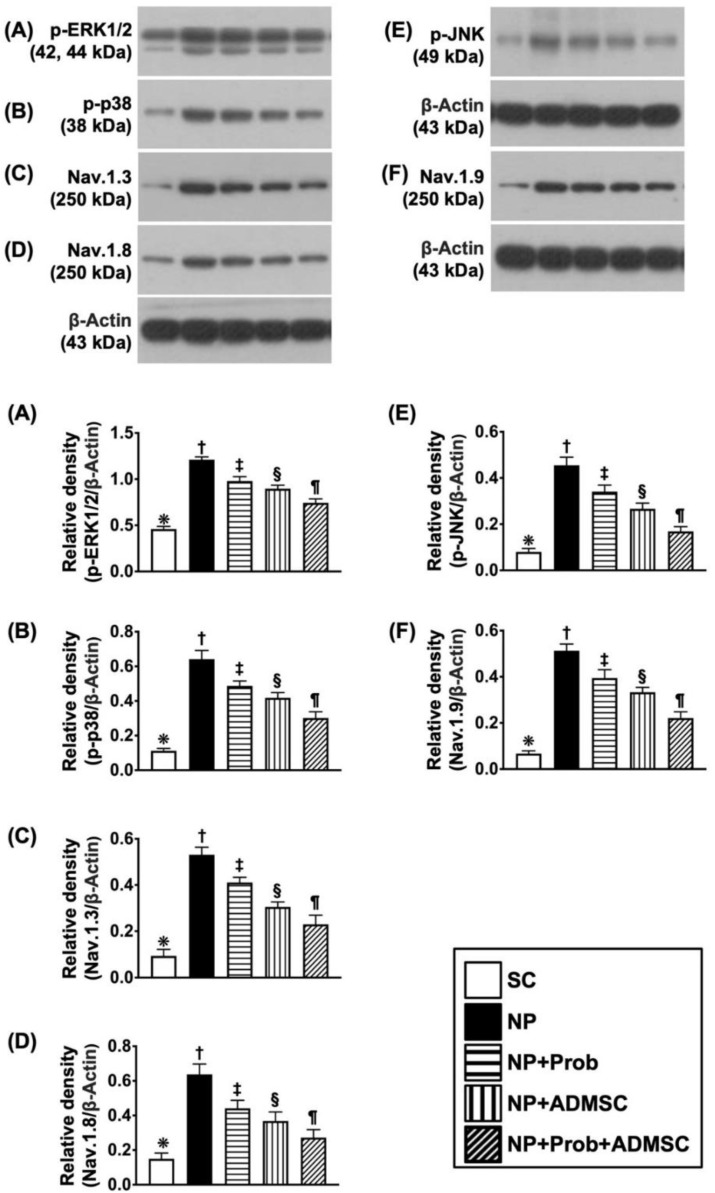
The protein expressions of mitogen-activated protein kinase (*MAPK*) family in L4-5 DRGs and voltage-gated sodium channels in sciatic nerves by day 60 after NP induction. (**A**) Protein expression of phosphorylated (p)-ERK1/2, * vs. other groups with different symbols (†, ‡, §, ¶), *p* < 0.0001. (**B**) Protein expression of p-JNK, * vs. other groups with different symbols (†, ‡, §, ¶), *p* < 0.0001. (**C**) Protein expression of p-p38, * vs. other groups with different symbols (†, ‡, §, ¶), *p* < 0.0001. (**D**) Protein expression of Nav.1.3, * vs. other groups with different symbols (†, ‡, §, ¶), *p* < 0.0001. (**E**) Protein expression of Nav.1.8, * vs. other groups with different symbols (†, ‡, §, ¶), *p* < 0.0001. (**F**) Protein expression of Nav.1.9 * vs. other groups with different symbols (†, ‡, §, ¶), *p* < 0.0001. Symbols (*, †, ‡, §, ¶) indicate significance for each other (at 0.05 level). All statistical analyses were performed using one-way ANOVA followed by Bonferroni multiple comparison post hoc test (n = 6 for each group). SC = sham control; NP = neuropathic pain; Prob = Probiotics; ADMSCs = adipose-tissue-derived mesenchymal stem cells.

To understand the activity of MAPK family biomarkers in NP, we utilized Western blot analysis again, and the result demonstrated that the protein expressions of p-ERK1/2 (Figure 5A), p-p38 (Figure 5B), and p-JNK (Figure 5E)—three mediators of extracellular signal of regulating kinases [14,46]—were lowest in group 1, highest in group 2, significantly higher in group 3 than in groups 4 and 5, and significantly higher in group 4 than in group 5. Additionally, the protein expressions of Nav.1.3 (Figure 5C), Nav.1.8 (Figure 5D), and Nav.1.9 (Figure 5F) in the sciatic nerve, three mediators of voltage-gated sodium channels [14,46], exhibited a similar pattern of MAPK family among the groups.

### 2.7. Cellular Expressions of Co-Localization between p-p38, p-EKR, and Peripherin and Co-Localization between Nav.1.8 and Peripherin in DRG Neurons

**Figure 6:**
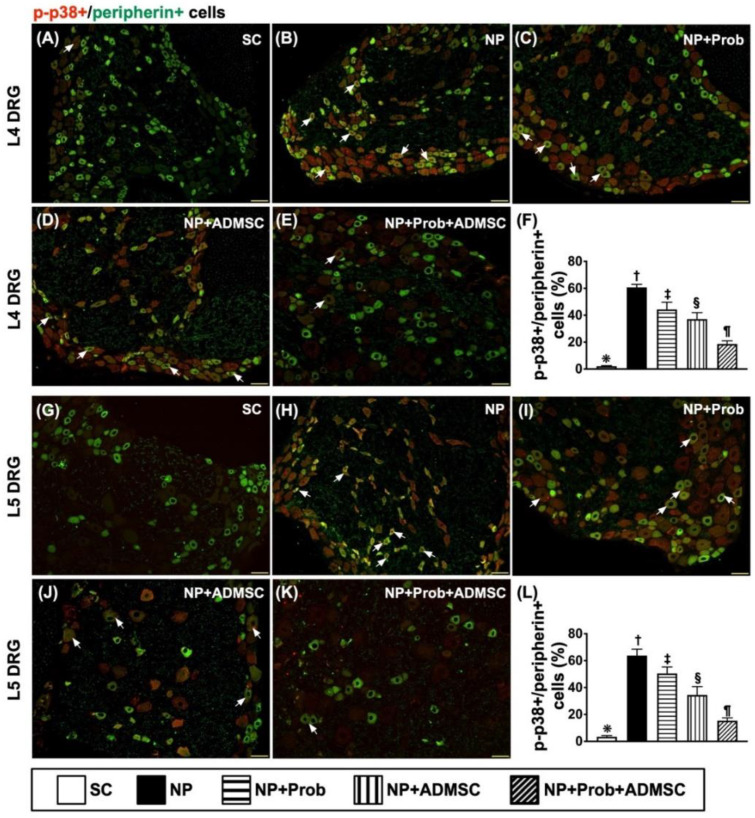
Cellular expression of co-localizations between p-p38 and peripherin in L4-L5 DRGs by day 60 after NP induction L4 DRG level: (**A**–**E**) Showing the immunofluorescent (IF) microscopic findings (200×) to identify merged positively stained p-P38 and peripherin (green–red co-localization). (**F**) Analytical results of number of p-P38+/peripherin+ cells, * vs. other groups with different symbols (†, ‡, §, ¶), *p* < 0.0001. L5 DRG level: (**G**–**K**) Showing IF microscopic finding (200×) for identifying merged positively-stained p-P38 and peripherin (green-red co-localization). (**L**) Analytical results of number of p-P38+/peripherin+ cells, * vs. other groups with different symbols (†, ‡, §, ¶), *p* < 0.0001. Note that the white arrows indicate the merged double stain of p-P38+/peripherin+. Scale bars in the lower-right corner represent 50µm. Symbols (*, †, ‡, §, ¶) indicate significance for each other (at 0.05 level). All statistical analyses were performed using one-way ANOVA followed by Bonferroni multiple comparison post hoc test (n = 8 for each group). SC = sham control; NP = neuropathic pain; Prob = probiotics; ADMSCs = adipose-tissue derived mesenchymal stem cells.

**Figure 7:**
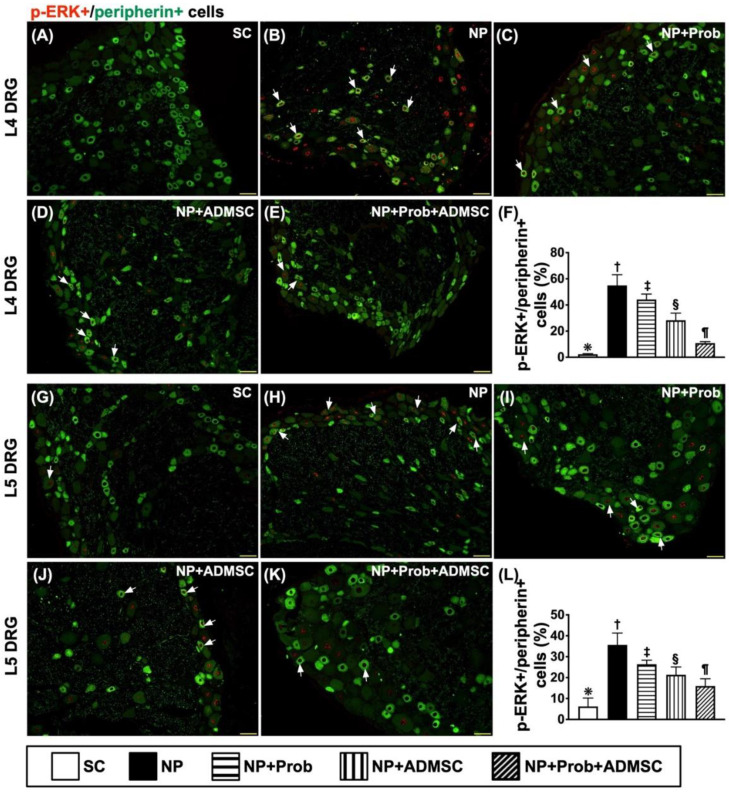
Cellular expression of co-localizations between p-EKR and peripherin in L4-L5 DRGs by day 60 after NP induction. L4-DRG level: (**A**–**E**) Showing the immunofluorescent (IF) microscopic findings (200×) to identify merged positively stained p-ERK and peripherin (green–red colocalization). (**F**) Analytical results of number of p-ERK+/peripherin+ cells, * vs. other groups with different symbols (†, ‡, §, ¶), *p* < 0.0001. L5-DRG level: (**G**–**K**) Showing IF microscopic findings (200×) to identify merged positively stained p-ERK and peripherin (green–red colocalization). (**L**) Analytical results of number of p-ERK+/peripherin+ cells, * vs. other groups with different symbols (†, ‡, §, ¶), *p* < 0.0001. Note that the white arrows indicate the merged double stain of p-ERK+/peripherin+. Scale bars in the lower-right corner represent 50 µm. Symbols (*, †, ‡, §, ¶) indicate significance for each other (at the 0.05 level). All statistical analyses were performed using one-way ANOVA followed by Bonferroni multiple comparison post hoc test (n = 8 for each group). SC = sham control; NP = neuropathic pain; Prob = probiotics; ADMSCs = adipose-tissue-derived mesenchymal stem cells.

The result revealed that the co-localization of p-P38 and peripherin in DRG neurons was highest in group 2, lowest in group 1, and significantly and progressively attenuated in groups 3 to 5, implying that the peripheral nerve injury and thermal and noxious stimuli were augmented in the NP that was most effectively suppressed by probiotic–ADMSC therapy (Figure 6A–L). Additionally, the IF microscopic findings also identified that co-localization between p-ERK and peripherin (i.e., the double staining), acting as a functional property of p-P38-peripherin, exhibited an identical pattern of co-localization to p-P38 and peripherin in DRG neurons among the groups (Figure 7A–L).

**Figure 8:**
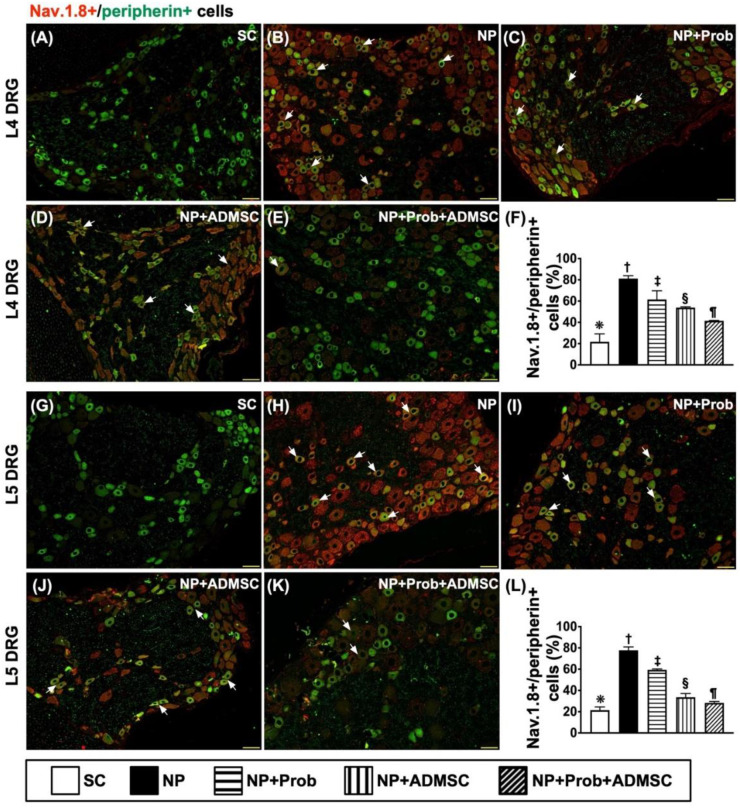
Cellular expressions of co-localizations between Nav.1.8 and peripherin L4-L5 DRGs neurons by day 60 after NP induction L4-DRG level: (**A**–**E**) Showing the immunofluorescent (IF) microscopic findings (200×) to identify merged positively stained Nav.1.8 and peripherin (green–red co-localization). (**F**) Analytical results of number of Nav.1.8+/peripherin+ cells, * vs. other groups with different symbols (†, ‡, §, ¶), *p* < 0.0001. L5-DRG level: (**G**–**K**) Showing IF microscopic findings (200×) to identify merged positively stained Nav.1.8 and peripherin (green–red co-localization). (**L**) Analytical results of number of Nav.1.8+/peripherin+ cells, * vs. other groups with different symbols (†, ‡, §, ¶), *p* < 0.0001. Note that the white arrows indicate the merged double stain of Nav.1.8+/peripherin+. Scale bars in the lower-right corner represent 50 µm. Symbols (*, †, ‡, §, ¶) indicate significance for each other (at the 0.05 level). All statistical analyses were performed using one-way ANOVA followed by Bonferroni multiple comparison post hoc test (n = 8 for each group). SC = sham control; NP = neuropathic pain; Prob = probiotics; ADMSCs = adipose-tissue-derived mesenchymal stem cells.

Furthermore, when we looked at the double stain of co-localization of Nav.1.8 and peripherin in the sciatic nerve, an indicator of the voltage-gated sodium channel serving as an ectopic discharge or activity in response to mechanical and thermal stimuli [14,46], exhibited a similar pattern of p-P38-peripherin in the sciatic nerve among the groups (Figure 8A–L).

### 2.8. Immunofluorescent Microscopic Evaluation for Identification of Co-Situation of p-P38 and NF200 in L4-5 DRG Neurons

**Figure 9:**
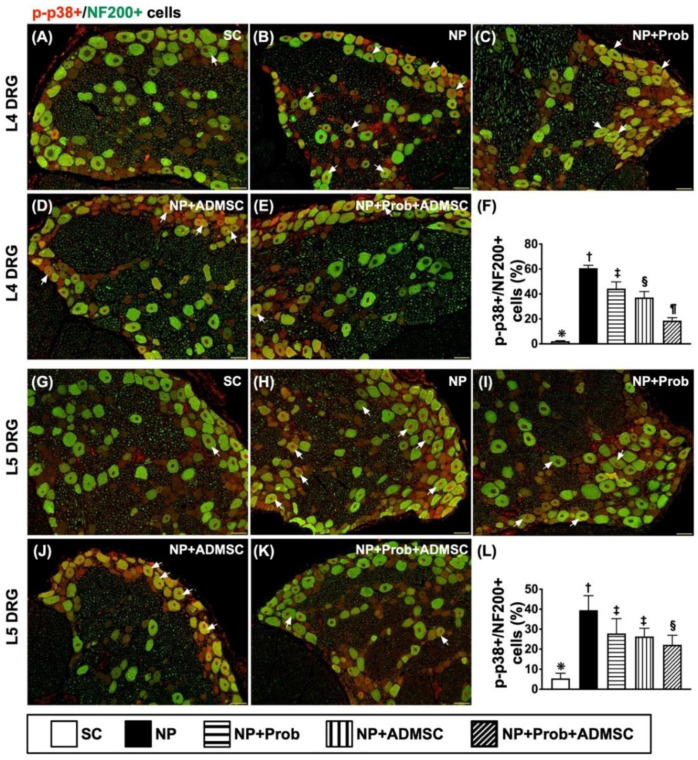
Immunofluorescent microscopic evaluation for identification of co-localization of p-P38 and NF200 in L4-5 DRGs neurons by day 60 after NP induction L4-DRG level: (**A**–**E**) Illustrating the immunofluorescent (IF) microscopic findings (200×) to identify merged positively stained p-P38 and NF200 (green–red co-localization). (**F**) Analytical results of number of p-P38+/NF200+ cells, * vs. other groups with different symbols (†, ‡, §, ¶), *p* < 0.0001. L5-DRG level: (**G**–**K**) Illustrating IF microscopic findings (200×) to identify merged positively stained p-P38 and NF200 (green–red co-localization). (**L**) Analytical results of number of p-P38+/NF200+ cells, * vs. other groups with different symbols (†, ‡, §, ¶), *p* < 0.0001. Note that the white arrows indicate the merged double stain of p-P38+/NF200+. Scale bars in the lower-right corner represent 50 µm. Symbols (*, †, ‡, §, ¶) indicate significance for each other (at the 0.05 level). All statistical analyses were performed using one-way ANOVA followed by Bonferroni multiple comparison post hoc test (n = 8 for each group). SC = sham control; NP = neuropathic pain; Prob = probiotics; ADMSCs = adipose-tissue-derived mesenchymal stem cells.

Next, we intended to elucidate both the small and large myelinated A-β fiber DRG neurons in NP induction in rats; IF microscopy was utilized again for identification of p-P38 expression in NF200. As we expected, the result revealed that the co-localization of p-P38 and NF200 in DRG neurons was highest in group 2, lowest in group 1, and then significantly and progressively attenuated in groups 3 to 5 (Figure 9A–L).

## 3. Discussion

The current study, which surveyed synergic effect of probiotics-facilitated ADMSC therapy on suppressing NP induced by CCI procedure in rodents, begot striking preclinical information. Frist, we successfully created a reproducible NP animal model that could contribute to other investigators interested in exploring various therapeutic modalities for the purpose of improving the clinical outcomes of NP. Second, this study elucidated that the impact of probiotic therapy on ameliorating NP was comparable to that of ADMSCs. Of importance was that probiotics-supported AMDMSCs were superior to just one treatment in alleviating NP, highlighting that there should be a favorably synergistic effect of this combination regimen on improving the outcome of NP in a clinical setting, especially for those patients who are refractory to conventional treatment. Finally, the results of this study distinctively clarified that not only the inflammation and oxidative stress but also the MAPK family medicators together participated in the maintenance of NP (i.e., certified by the time courses of TPWL and MPWT expressions in living rodents).

Most NP patients frequently present a suffering appearance due to the absence of an effective treatment for chronic intractable NP, resulting in poor spirits, emotional exhaustion, recurrent insomnia and depression, and irritability in every moment. The most important discovery in the current study was that the thresholds of TPWL and MPWT, which were remarkedly reduced in NP animals, were substantially reversed by probiotics, further substantially reversed by ADMSCs, and further substantially reversed by the combination therapy of probiotics-ADMSCs. Importantly, when observing the time courses of the TPWL and MPWT examinations, we found that these regimens upregulated the TPWL and MPWT thresholds not only in the early stage (i.e., by day 7) but also persistently into the late stage (i.e., by day 60) of NP, highlighting that these therapeutic modalities, especially in the regimen of probiotics–ADMSCs, could offer great impact on treating the chronic setting of NP, especially in NP patients who are refractory to traditional therapy. Additionally, our data also supported that early treatment would be the fundamental requirement for offering great benefits to the NP patients.

It is recognized that the underlying mechanistic basis involved in NP is connected to the secondary nociceptive stimulus with actual or potential tissue damage from inflammation and immune activation [11,12,13,14,15,16,17,47]. A crucial finding in this study was that not only the damaged zone but also the systemic inflammatory reactions were markedly upregulated in NP animals as compared to the controls. Additionally, the stress-signaling cascades of the MAPK family and the facilitated pain-transmitting mediators of voltage-gated sodium channels (i.e., Nav.1.3, Nav.1.8, and Nav.1.9) were also substantially augmented in NP animals as compared to the controls. However, these aforementioned molecular–cellular perturbations were substantially reversed by probiotics or ADMSCs and further substantially reversed by combined probiotics and ADMSC treatment in NP animals. In this way, our results, in addition to extending the results of previous studies [11,12,13,14,15,16,17,47], could cardinally explain why pain thresholds (i.e., the thresholds of TPWL and MPWT) and the outcomes of NP animals were notably improved after receiving probiotic–ADMSC therapy.

Only limited studies have previously reported that cell-based therapy could serve as an innovative therapy for painful peripheral neuropathy [48,49]. On the other hand, fewer studies have revealed that probiotic therapy would act as a possible adjuvant regimen for counteracting NP [24]. A principal finding in this study was that probiotic or ADMSC treatment notably and comparably preserved the functional integrity of peripheral nerves, i.e., upregulated the hyperalgesia threshold, resulting in augmentation of the analgesia of NP. In this way, our finding reinforced the findings of previous research [24,48,49]. Of innovative finding in the present study was that combination treatment with probiotics and ADMSCs could even offer a synergic effect on upregulating the thresholds of TPWL and MPWT (i.e., indicated by the pain threshold that was defined as the point beyond which a stimulus caused pain), suggesting that our findings extended the findings of previous investigations [24,48,49].

Links between tissue damage and generations of cell apoptosis, mitochondrial dysfunction and DNA damage have been keenly investigated by numerous studies [13,14,15,16,17,25,26,27]. Of importance is that these damaged tissues which elicited biomarkers would, in turn, participate in upregulation of the inflammatory reaction and oxidative stress and worsen the painful sensation [13,14,15,16,17,25,26,27]. An essential finding in the present study was that the apoptotic and DNA-damaged biomarkers were markedly increased in NP animals as compared to SC animals. However, these parameters were notably suppressed by probiotic or ADMSC treatment and further notably suppressed by combined probiotic and ADMSC treatment, once again suggesting that probiotics facilitated the ADMSC treatment’s ability to improve the outcomes of NP animals.

The readers would be interested in knowing why probiotic–ADMSC therapy would offer a synergic effect for alleviating NP in rodents. We proposed that there could be at least two reasons explained for the synergic effect of this combined regimen. First, abundant data have testified that the MSCs have inherent abilities of suppression of inflammation [25,26,27] and innate and adaptive immunity, augmentation of tissue regeneration, and secretion of neural trophic factors [25,26,27,28]. Second, on the other hand, studies have indicated that gut microbiota play a crucial role in many types of chronic pain, including inflammatory pain, headache, neuropathic pain, and opioid tolerance [20,21], as well as immune dysregulation [22,23]. However, gut microbiota which induce the above-mentioned perturbations [20,21,22,23] are usually effectively suppressed by probiotics treatment [24].

Study Limitation

This study has limitations. First, personalized probiotics which could offer greater benefit for suppressing NP were not utilized for rodents. Second, we did not directly investigate the impact of the rodent’s gut microbiota on the brain–peripheral nerve axis and test the crosstalk between alteration of gut microbiota and peripheral neuropathy after probiotic treatment. Third, numerously studies have also shown that mouse models of fecal microbiota were also a reliable modality for the investigation of different disease entities. Without comparison between rat and mouse models of NP, we did not know whether the rat model was better than the mouse model for investigating the impact of probiotic and ADMSC therapy on attenuating CCI-induced NP or vice versa.

## 4. Materials and Methods

### 4.1. Ethical Statement

All animal procedures were approved by the Institute of Animal Care and Use Committee at Kaohsiung Chang Gung Memorial Hospital (Affidavit of Approval of Animal Use Protocol No. 2019102405).

### 4.2. To Create an Animal Model of Neuropathic Pain

Left sciatic mononeuropathy in each rat was induced by a chronic constriction injury (CCI) procedure which has been described in our previous report [14]. Briefly, animals in each group were anesthetized via inhalation of 2.0% isoflurane for laminectomy. The left sciatic nerve was exposed and carefully isolated. Four chromic gut ties (4–0) were used to ligate the nerve at 1 mm intervals loosely enough so that, under microscopic inspection, the epineural blood flow was not blocked. The incision was then closed in layers, and rats were allowed to recover from anesthesia. Sham surgical intervention was conducted for exposing the sciatic nerve without performing the ligations.

### 4.3. Behavioral Evaluation for MPWT and TPWL

The procedure and protocol have been reported in our previous study [14]. In detail, to investigate the impact of ADMSC–probiotic treatment on suppressing the NP at acute, subacute, and chronic stages, the thermal and mechanical nociceptive thresholds were assessed prior to CCI and on days 1, 7, 14, 28, 42, and 60 after CCI procedure.

To assess thermal hyperalgesia, animals were placed on a glass plate and radiant heat (Plantar Test Apparatus; Ugo Basile, Gemonio, Italy) was conducted to the plantar surface of the surgical hind paw. The withdrawal latency and duration were documented, with a minimum value set at 0.1 s and a cutoff latency set at 30 s to prevent paw damage. Each animal was examined a total of three times at intervals of 5 min, and the mean value was utilized in the following data analysis.

To evaluate the mechanical allodynia, each rat was placed in the chamber, and a servo-controlled mechanical stimulator (Dynamic Plantar Aesthesiometer; Ugo Basile, Gemonio, Italy) was exerted on the plantar surface of the operated hind paw repeatedly at 5 min intervals with gradually enhancing punctate pressure until the animal withdrew its paw. A maximal cutoff value was set at 50 g to prevent paw damage. The threshold was examined thrice for each time point, and mean values were utilized for the analysis.

### 4.4. Animal Grouping

Sprague–Dawley rats (n = 50) weighing 300–325 g were utilized for the present study. Animals were equally divided into group 1 (Sham-operated control (SC), i.e., dissected the skin and muscle layers and merely isolated the sciatic nerve, followed by closing the skin and muscle layers), group 2 (NP induced by CCI procedure), group 3 (NP + probiotics (i.e., 1.5 billion colony-forming units (CFU) for each rat orally at 3 h after CCI procedure, followed by QOD fir 30 days, i.e., up to the end of study)), group 4 (NP + ADMSCs (3.0 × 10^5^ cells) by intravenous administration at 3 h after CCI procedure, followed by QOD × 6 times (i.e., 7 times in total to mimic the clinical setting of drug use)) and group 5 (NP + probiotics + ADMSCs, i.e., the dosages of these two therapies as in groups 3 and 4). Animals in each group were euthanized at day 60 (i.e., for evaluation of the long-term outcomes) after CCI procedure, and the ipsilateral sciatic nerve and L4-L5 DRGs were harvested for individual study. Additionally, the procedure and protocol of experimental strategy were schematically illustrated in Supplementary Figure S2.

### 4.5. Isolation of Autologous ADMSCs for the Study

By day 14 prior to CCI procedure, isolation of adipose tissue for culturing the ADMSCs was conducted in animals of groups 4 and 5. Adipose tissues around the epididymis and abdomen were cautiously harvested based on our previous reports [26,27]. After 14 days of cell culture, approximately 2.5–3.5 × 10^6^ ADMSCs were obtained from each rat.

### 4.6. The Characteristics of Probiotics to Be Utilized in the Present Study

Based on our preliminary data (refer to Supplementary Table S1), we found the Lacinobacter B was the most popular gut microbiota in the rats (evaluated from the stool of two rats). On the other hand, the LP3 probiotic is the most effective probiotic for inhibiting inflammation and reactive oxygen species and regulating the immune system (i.e., increasing Th1 and downregulating Th2). Therefore, personalized probiotics, i.e., named LP3 probiotics, were utilized in the present study.

### 4.7. Method for Microbiome Analysis

Genomic DNA was extracted using a Presto™ Soil DNA Extraction Kit (Geneaid, New Taipei City, Taiwan) according to the manufacturer’s instructions for stool samples. An equivalent of 1 mg of each sample was used for DNA quantification using a Qubit 4 fluorometer with the dsDNA broad range assay (Invitrogen, Waltham, MA, USA). DNA integrity was examined via microcapillary electrophoresis using a LabChip GX Touch with the DNA High Sensitivity Assay (Perkin-Elmer, Waltham, MA, USA).

### 4.8. Immunofluorescent (IF) Staining

The procedure and protocol of IF staining have been described in our previous reports [14,15,26]. Briefly, rehydrated paraffin sections were first treated with 3% hydrogen peroxide (H_2_O_2_) for 30 min and incubated with Immuno-Block reagent (BioSB, Santa Barbara, CA, USA) for 30 min at room temperature. Sections were then incubated with primary antibodies specifically against phosphorylated (p)-p38 (1:200, GeneTex, Irvine, CA, USA), NF200 (1: 200, Sigma-Aldrich, St. Louis, MO, USA), peripherin (1:1000, Abcam, Cambridge, UK) 53BP1 (1:700, Novus, Littleton, CO, USA) and β3 Tubulin (1:200, Santa Cruz, Dallas, TX, USA). Sections were incubated with irrelevant antibodies serving as controls. Three sections of DRG specimens were analyzed in each rat. For quantification, three randomly selected high-power fields (HPFs) were analyzed per section. The mean number of positively stained cells per HPF for each group of the animals was analyzed across all nine HPFs.

### 4.9. Western Blot Analysis

The ipsilateral sciatic nerve, L4-L5 DRGs, and corresponding dorsal horn of the spinal cord from the rats of the sham control and experimental groups were harvested as previously described [14,15,26]. Equal amounts (50 µg) of protein extracts were loaded and separated via SDS-PAGE using 8–12% acrylamide gradients. After electrophoresis, the separated proteins were transferred electrophoretically to a polyvinylidene difluoride (PVDF) membrane (Amersham Biosciences, Amersham, UK). Nonspecific sites were blocked via incubation of the membrane in blocking buffer (5% nonfat dry milk in T-TBS (TBS containing 0.05% Tween 20)) overnight. The membranes were incubated with the indicated primary antibodies (refer to Supplementary Table S2) for 1 h at room temperature. After electrophoresis, the separated proteins were transferred electrophoretically to a PVDF membrane (Amersham Biosciences, Amersham, UK). The raw materials, i.e., original uncropped and unprocessed scans of the blots in the sections of original images of blots and gels, were provided as per Supplementary Figure S3.

Nonspecific sites were blocked via incubation of the membrane in blocking buffer (5% nonfat dry milk in T-TBS (TBS containing 0.05% Tween 20)) overnight.

### 4.10. Statistical Analysis

Quantitative data are expressed as mean ± SD. Statistical analysis was performed using ANOVA followed by Bonferroni multiple comparison post hoc test. SAS statistical software for Windows version 8.2 (SAS Institute, Cary, NC, USA) was utilized. A probability value <0.05 was considered statistically significant.

## 5. Conclusions

Combined probiotic and ADMSC therapy, which was a novel idea for treatment of NP, provided an innovative and promising way for counteracting NP in rodents.

## Data Availability

The data contained within the paper are available from the authors upon reasonable request.

## Supplementary Materials

The following supporting information can be downloaded at: https://www.mdpi.com/article/10.3390/ijms231911974/s1.
