# Supplementary material for: Synergic Effect of Early Administration of Probiotics and Adipose-Derived Mesenchymal Stem Cells on Alleviating Inflammation-Induced Chronic Neuropathic Pain in Rodents
Source: Int J Mol Sci. 2022 Oct 9;23(19):11974. doi: 10.3390/ijms231911974 (PMC9570240; doi:10.3390/ijms231911974)
Supplement: Supplementary file 1 [file ijms-23-11974-s001.zip › ijms-1930613-supplementary.pdf]

Figure S1. Relative abundances of microbiome analysis based on Taxonomic profile

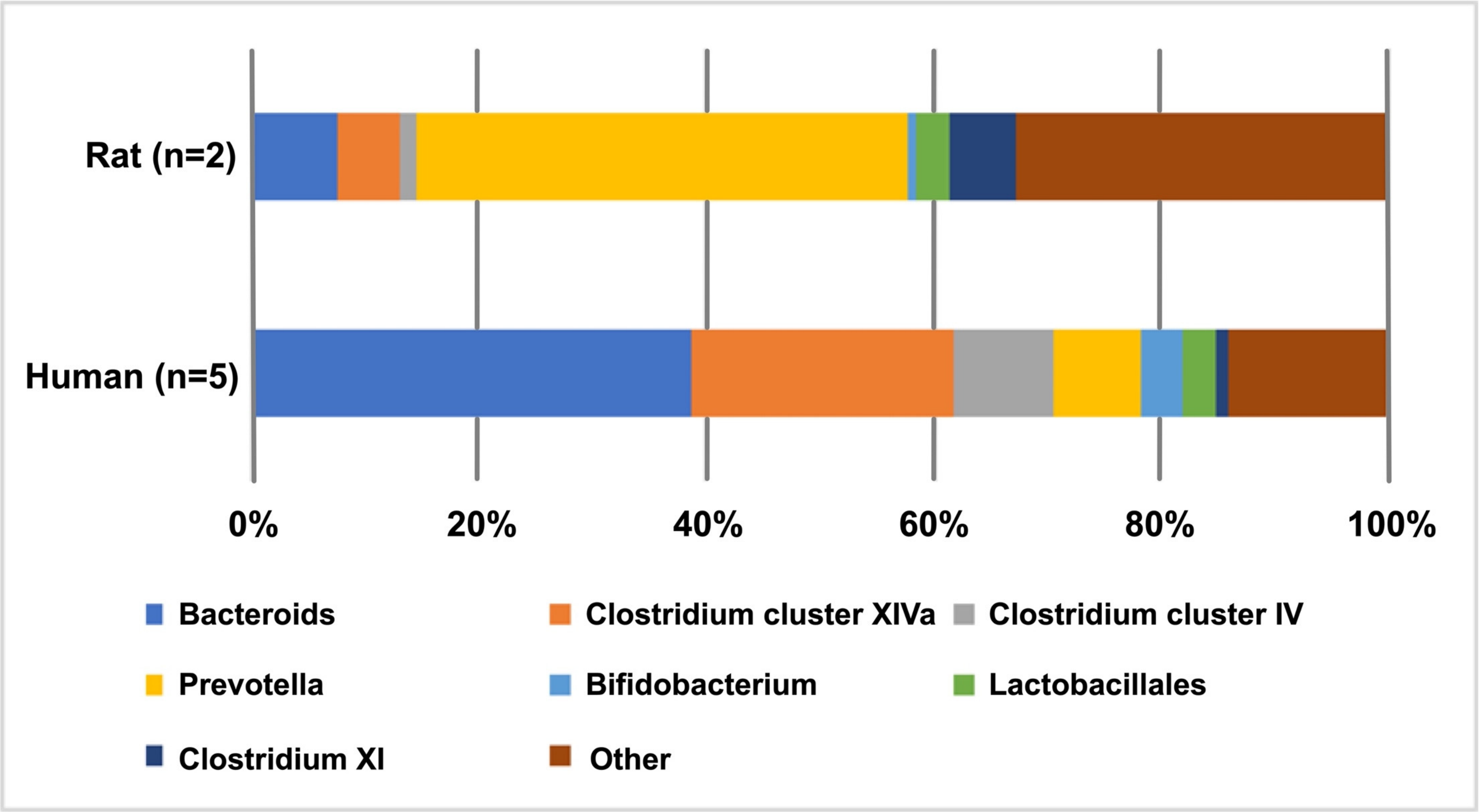

Figure S2. Procedure and protocol of experimental strategy

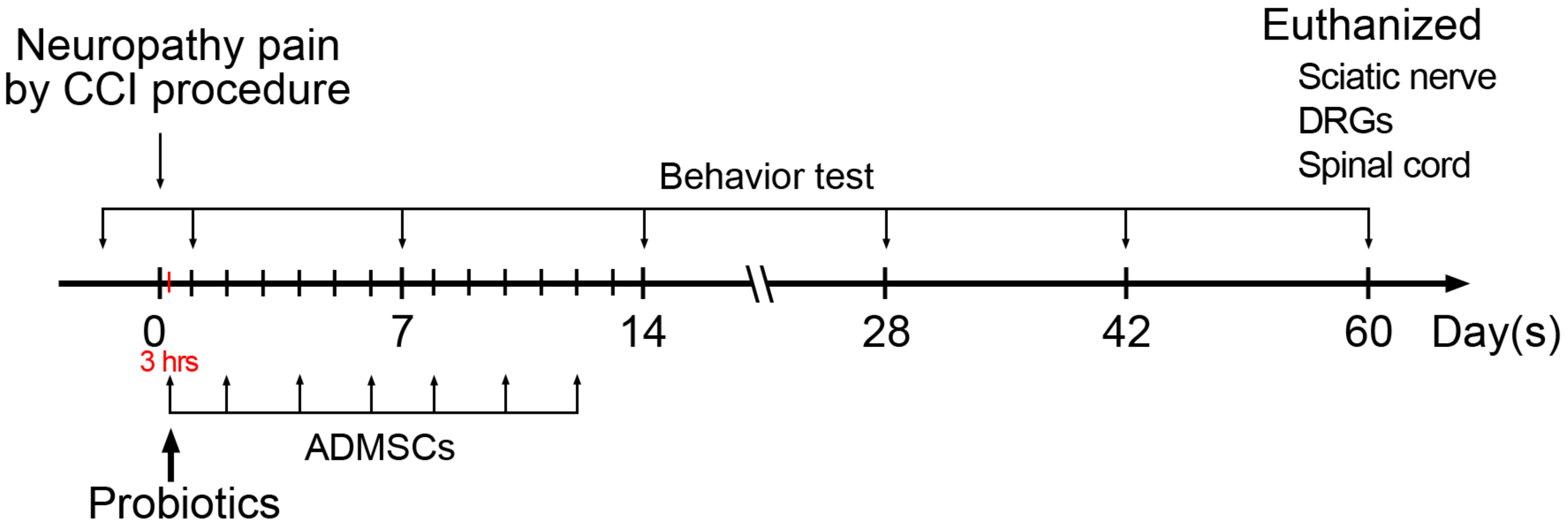

Table S1. Each percentage of microbiome analysis in healthy people and rats

	Heathy people (n=5)	Heathy rat (n=2)
Bacteroides (%)	38.75	7.48
Clostridium cluster XIVa (%)	23.16	5.44
Clostridium cluster IV (%)	8.77	1.63
Prevotella (%)	7.62	43.25
Bifidobacterium (%)	3.48	0.78
Lactobacillales (%)	3.14	2.79
Clostridium XI (%)	1.23	5.76
Others (%)	13.85	32.87
Total (%)	100	100

Table S2. Antibodies used for immunofluorescence (IF) and Western blot analyses

Product name	Concentration, catalog number code and supplier
**Western blot antibodies**	
phosphorylated (p) nuclear factor (p-NF)-κB	(1:1000, Cell Signaling, #3033, Danvers, Massachusetts)
interleukin (IL)-1ß	(1:1000, Cell Signaling, #12426, Danvers, Massachusetts)
tumor necrosis factor (TNF)-α	(1:1000, Cell Signaling, , #3707, Danvers, Massachusetts)
matrix metalloproteinase (MMP)-9	(1:1000, Abcam, ab76003, Cambridge, UK)
NOX-1	(1:1000, Sigma, SAB4200097, Massachusetts, USA)
NOX-2	(1:1000, Sigma, SAB4200118, Massachusetts, USA)
cleaved (c) caspase 3	(1:1000, Cell Signaling, #9665, Danvers, Massachusetts)
c-Poly (ADP-ribose) Polymerase (C-PARP)	(1:1000, Cell Signaling, #9542, Danvers, Massachusetts)
γ-H2AX	(1:1000, Cell Signaling, #7631, Danvers, Massachusetts)
phosphorylated extracellular signal-regulated protein kinase (p-ERK1/2)	(1:3000, Millipore, 442685, Burlington, MA, U.S)
phosphorylated c-Jun N-terminal kinase (p-JNK)	(1:1000, Abcam, ab4821, Cambridge, UK)
p-p38	(1:1000, Sigma, M8177, Massachusetts, USA)
Nav.1.3	(1:500, Alomone, ASC004, Jerusalem, Israel)
Nav.1.8	(1:500, Abcam, ab63331, Cambridge, UK)
Nav.1.9	(1:500, Alomone, ASC017, Jerusalem, Israel)
horseradish peroxidase-conjugated anti-rabbit immunoglobulin IgG	(1:5000, Sigma, A0545, Massachusetts, USA)
**Immunofluorescent (IF) antibodies**	
phosphorylated (p)-p38	(1:200, GTX50280, Gene Tex, Irvine, CA)
NF200	(1:200, NO142, Sigma-Aldrich, Massachusetts, USA)
peripherin	(1:500, ab106276, Abcam, Cambridge, UK)
53BP1	(1:1000, NB100-304, Novus, Colorado, USA)
β3 Tubulin	(1:200, SC-51670, Santa Cruz, Texas, USA)
